# Pharmaceutical workers’ perceptions of physical activity and healthy eating: a qualitative study

**DOI:** 10.1186/s13104-021-05765-8

**Published:** 2021-09-08

**Authors:** Philippe Jean-Luc Gradidge, Catherine E. Draper, Daleen Casteleijn, António Palmeira

**Affiliations:** 1 Centre for Exercise Science and Sports Medicine, Faculty of Health Sciences, University of the Witwatersrand, Johannesburg, South Africa; 2 SAMRC/Wits Developmental Pathways for Health Research Unit, Department of Paediatrics, School of Clinical Medicine, Faculty of Health Sciences, University of the Witwatersrand, Johannesburg, South Africa; 3 Occupational Therapy Department, School of Therapeutic Sciences, Faculty of Health Sciences, University of the Witwatersrand, Johannesburg, South Africa; 4 CIDEFES, University Lusóphone, Lisbon, Portugal; 5 Executive Director, International Society of Behavioral Nutrition and Physical Activity (ISBNPA), Minneapolis, MN United States of America

**Keywords:** Physical activity, Diet, Worker, African, Qualitative study

## Abstract

**Objective:**

The public health message ‘move for health’ is relevant given the high prevalence of insufficient physical activity, particularly in African countries. The call for behaviour modification including limiting unhealthy dietary patterns in these settings is therefore critical; however, there is limited knowledge on the adoption of health promotion strategies in the workplace. This study aimed to investigate workers’ perceptions of physical activity and healthy eating.

**Results:**

Five focus groups were conducted with 28 participants employed in a South African pharmaceutical manufacturing company to explore perceptions of physical activity and healthy eating. Results showed that two categories emerged: physical activity and unhealthy behaviours. Participants recognised the importance of obtaining sufficient physical activity in various domains, however believed that contemporary lifestyle limited opportunities for movement. Likewise, participants viewed healthy eating as unrealistic due to financial constraints. There was however agreement that total physical activity time could be increased during recreational pursuits outside of vocational time and may include intermittent walking for travel. These findings are important for workplace interventions and provide a more robust understanding of workers’ perceptions of physical activity and healthy eating.

**Supplementary Information:**

The online version contains supplementary material available at 10.1186/s13104-021-05765-8.

## Introduction

Chronic, non-communicable diseases (NCDs) remain a major burden in low- and middle-income countries (LMICs) such as South Africa, where 51% of all mortality had an NCD-related origin in 2016 [1]. Obesity is a complex disease with an aetiology in lifestyle behaviours, and closely associated with the manifestation of NCDs. A 2018 repeated panel study of South African adults showed that the prevalence of obesity increased from 23.5% in 2008 to 27.2% in 2012 [2], and is predicted to continue increasing [3]. Low-income populations living in urban settings are particularly vulnerable to obesity as unhealthy eating practices and physical inactivity behaviours are adopted with rapid urbanisation. The dietary pattern of urban South Africans lacks nutrient variation and is associated with obesity-related diseases, comprised of saturated fats, energy-dense street foods, and fruits and vegetables. Physical inactivity is also a concern and now considered a global public health problem [4]. In South Africa, 37% of the population are not meeting physical activity guidelines and urban populations are less active than rural-dwelling South Africans [5]. The emergence of obesity-related NCDs is also a burden in the South African workforce as studies demonstrate a high prevalence of preventable diseases and, associated risky lifestyle behaviours in employees across several corporate sectors [6, 7].

Physical activity can offset the risk factors associate with NCDs and improve the overall health of workers [8]. Short-term improvements in weight and blood pressure were observed in female employees of a South African university following a 12-week walking programme [9]. A longitudinal two-year lifestyle intervention study showed similar positive changes in physical activity, diet, blood pressure and cholesterol in workers at a South African power plant [10]. Organisational support, acceptance from employees, and tangible employee benefits were noted as important components of long-standing success of this intervention [10]. Further research on sustainable workplace programmes is required across various sectors to align with the concept of a healthy South African workforce [11].

Limited evidence has explored pharmaceutical manufacturing employees’ perceptions of physical activity and healthy eating in LMICs. Therefore, the aim of this study was to describe how South African employees working in this industry viewed their own behaviours in the context of the working environment, commuting to work and their lived experiences at home.

## Main text

### Methods

#### Setting and sample

The setting of this qualitative study was a pharmaceutical manufacturing company located in an industrial region of Johannesburg, South Africa. The company operates 24 h/day, 7 days/week, manufacturing products for sale in South African and international pharmacy outlets.

Eight focus groups, each with 6–8 participants were planned. The study sample included a mixed group of 28 males and females from 200 employees, recruited by telephone, employed at least on a 50% workday at the company, representing persons from the various occupation types including administrative office work, production operators, pharmacy and laboratory work, cleaning and security staff, and quality assurance. Purposive sampling was used with inclusion criteria of ≥ 18 years old and willing to share their opinions on personal reasons for obesity and barriers and facilitators of physical activity. Ethical approval was obtained from the University of the Witwatersrand and all participants who provided written consent were included in the study.

#### Data collection

Focus groups were conducted by PJG (PhD), a male researcher and clinical professor with experience in conducting qualitative interviews and, observed by a female independent researcher with a PhD with experience in conducting qualitative interviews. PJG was unknown to the participants and had no previous relationship with them. An interview guide was designed to stimulate discussion of the participants’ views on physical activity and diet (Additional file 1). The focus groups continued until data saturation, which was reached after five focus groups, each 45–60 min long. All focus groups were audio recorded and transcribed verbatim by an independent transcriber.

#### Data analysis

Transcribed data were anonymised and exported into Atlas.ti 9 (ATLAS.ti Scientific Software Development GmbH). A contemporary approach to quality qualitative research was applied to this study [12]. Coders (PJG and DC) used a thematic content analysis approach with inductive open coding to determine categories and sub-categories from the focus group discussions [13]. The coders proceeded to name these categories using actual participant data. CED reviewed the process to ensure reliability and correct interpretation of the focus group discussions. All authors were responsible for the rigor and, any potential uncertainties and biases were noted and settled through discussion to maintain credibility and validity throughout the research process (Additional file 2).

### Results

Focus group participants (53.6% female, aged 27–59 years old) represented 5 groups of occupation types in the pharmaceutical manufacturing company (highest participation rate; administrative office work (32%), production staff (32%), pharmacy and laboratory workers (21%), cleaning and security staff (7%) and quality assurance (7%). Focus groups participants worked in the company for an average 3.82 years and had an age range 20–60 years old. The analysis resulted in two categories including 1) physical activity (Table 1) and 2) dietary behaviours (Table 2) and associated sub-categories.

**Table 1:** Focus group sub-categories for physical activity

Sub-category	Example excerpts
Personal barriers to physical activity	Now, responsibilities in life has changed. So I think that is as life changes your activity levels change as well as including your, health wise. I can't run ten blocks like before. (Participant ID: 1:90) If I can add, I don't know, like my growing up nè. I am the first born at home so I used to do washing for my younger sister and my brothers. (Participant ID: 1:86) Now it’s raining, it’s cold. Now, it’s getting cold now. (Participant ID: 2:90)
Transport to work	It’s almost the same, two hours one way coming here. In the morning, well, I do have a little bit of walk to the bus stop. (Participant ID: 2:12) Well, not much because the bus, you don’t feel ok standing in the bus, even if you try to change there is nothing you can change. (Participant ID: 2:15)
Physical activity at home	Like for me yes, because my son is only almost two years, so I have to run after him. Some bath, feeding, I have to run, and you have to fight for him to eat. (Participant ID: 2:27) Me sometimes I do a weightlifts. Yes maybe twice a week. Just to refresh my body. (Participant ID: 3:55) So, I agree with him that you have to create time and do individual exercises. (Participant ID: 2:47)
Culture	In some instances I think it goes worse because some, their husband they cannot help them because remember people with different cultures and how we see things, some women when they go home, they do everything. (Participant ID: 2:79) And if you are not open-minded in terms of the culture, you will struggle…. that is why I can argue that ladies they go through a lot of depression because of home. (Participant ID: 42:38)

**Table 2:** Focus group sub-categories for dietary behaviours

Sub-category	Example excerpts
Food and drink purchasing	It's that time of the month where you cannot afford it [healthy food]. But we will get paid today, and two days after, there is nothing. (Participant ID: 1:105) No I am not using it [the canteen], I don't have the money to pay for that food. (Participant ID: 1:101) You can't afford to buy [healthy food]. It's because of the salary. Yes. I don't have the money. And we are tired. (Participant ID: 1:114) I can't afford to buy myself an apple or maybe 100% juice. (Participant ID: 1:111)
What people eat	Every time in the morning, each one is having a fatcake [“magwinya”, a popular energy-dense, doughnut-type South African street food] (Participant ID: 3:98) It is not the junk food so much actually. It's vegetable foods. (Participant ID: 3:41)
Reasons for obesity	And if you come in check the women in packing, the majority of the women it's, they are overweight because it's from the canteen eating, and coming back to the line you know and working. (Participant ID: 1:57) It’s food because all this junk that we eat because remember, we absorb more fats, and we do not burn them. Remember, if you stop smoking, it is good, but if keep on eating junk and you stop smoking, you see now you are inviting something else. (Participant ID: 2:59)
Cigarette smoking	I feel, not exactly that I feel my chest, I feel like there is something that says no this thing is bad for my health. You understand. (Participant ID: 4:66) I used to be a smoker. I used to gym now I can’t. (Participant ID: 2:66)

#### Physical activity

##### Personal barriers to physical activity

The main view of participants was that physical activity improved health and protected against disease. Participants commonly described being previously engaged in moderate-to-vigorous physical activity, however with increasing family and household tasks, participants noted difficulty with maintaining an exercise regimen. Worsening weather conditions were perceived as reasons for not engaging in physical activity.

##### Transport to work

Despite a shared belief among participants concerning the health benefits of active transportation, converting this belief into actionable behaviours presented with many difficulties. One such difficulty discussed by participants was a belief that transport to work promoted sedentary travel as most commuted for more than 400 min/day. It was suggested that walking as means of active travel was within reach as some participants walked to the bus stop to wait for public transport.

##### Physical activity at home

Although many participants perceived the home environment and associated responsibilities as challenging for maintaining active lifestyle, some commented that unstructured movement in the home was obtained during child-care. Others believed that they were engaged in resistance training at home by using free-weights. Some participants suggested constructing workplace exercise facilities, however the majority of the participants felt that this was impractical and viewed leisure-time physical activity as a more realistic option.

##### Culture

The participants described their concerns around cultural factors, indicating that women were expected to cook and clean the house, while men remained inactive at home. There was agreement that this cultural norm increased women’s stress and resulted in lower opportunities for physical activity because they were expected to serve their spouses and families after returning from work. Overall, participants emphasized that domestic responsibilities restricted healthy behaviour.

#### Dietary behaviours

##### Food and drink purchasing

Most participants could correctly distinguish between unhealthy foods such as fried chips and healthier options such as fruits and vegetables, but the consensus amongst participants was that although an onsite canteen was available with both options, most were reluctant to purchase foods from this vendor due to limited availability of funds.

Many participants perceived that financial constraints hindered their opportunities to consume healthy foods and become physically active. In fact, some participants emphasized that their low incomes increased stress and depressive symptoms because of monthly payments with little disposable monies, which further limited the adoption of public health messaging. Several of those participants experiencing heightened stress shared that they viewed alcohol consumption as a coping mechanism.

##### What people eat

In keeping with the idea of healthy eating, participants believed that due to occupational demands in a 24-h coverage company and engaging in rotating shift work, their sleep quality and efficiency was low and resulted in poor eating behaviour. As a result, participants felt that this was the reason for purchasing convenient, energy rich foods on their commute to work. On the other hand, some participants felt that it was important to also consume more nutritious foods such as vegetables and fruits.

##### Reasons for obesity

Although most participants appreciated the importance of lifestyle behaviours for the prevention of weight gain, some believed that their sedentary job tasks and the consumption of energy dense foods were the primary reasons for the increasing presence of overweight and obesity. It was also believed that staff could reduce weight gain by reducing the intake of fatty foods. Some participants believed that quitting cigarette smoking affected obesity, and preferred to continue smoking to prevent weight gain.

##### Cigarette smoking

Active smokers in the focus group discussions also believed that support systems were important for cessation because previous self-imposed attempts had been unsuccessful. The concept of social support was believed to be important for adoption and maintenance of behaviour modification, however participants viewed physical activity after cessation as impractical and confirmed a desire to continue smoking regardless of the long term adverse effects on cardiovascular health and exercise capacity.

### Discussion

This study provides deeper insight into the challenges of maintaining healthy behaviours amongst workers in one South African pharmaceutical manufacturing industry. The key finding of this study was that most of the participants in our study understood the benefits of healthy behaviours, but believed that this was not financially sustainable in the context of food insecurity and healthy food availability. Cultural factors were perceived as influencers of sedentary behaviour. The general perception of healthy lifestyle was negative and highlights the urgent need for guiding health education in the workplace.

Physical activity is one of the main contributors of health in workers, but has received little attention in South Africa. This behaviour, in particular, the concept of shifting from none to at least some movement [8], can be accumulated during work hours. The prevailing notion among our study population was the physical activity at work was not possible because of limited opportunities for energy expenditure in sedentary occupations. The prevalence of insufficient physical activity in South African workers is higher than community estimates and ranges from ~ 64% to 77% [6, 14]. Previous South African [9, 10] and international studies [15] confirm that the physical activity during working hours improves the cardiovascular and mental health of employees. In our study, workers valued the role of physical activity promotion in the workplace and assigned great importance on lowering time spent in sedentary activities. Research indicates that this can be envisaged by maximising on opportunities for unstructured movement in the workplace and during active commuting as suggested by the participants [8].

A major concern that was raised was the fact that although consuming nutritious foods was viewed as important for health, most participants perceived their own financial constraints as a driver of poor eating behaviour. These findings are in support of earlier studies showing poor nutrition and diet variety in low-income South African workers [6, 16, 17]. A recent two-year intervention study in South African had promising results, demonstrating that nutritious foods could be subsidised and sold at a reduced cost through healthy eating initiatives at work [10]. The proportion of participants consuming inadequate fruits and vegetables was reduced and a significant drop in metabolic disease risk factors was observed. Despite these positive findings, the data may not be transferable to other South African worksites and further investigation is needed.

A solution such as The ‘*Capability, Opportunities, Motivation and Behaviour’* model can be applied to these contexts [18]. For example, while workers in our study stated that they were knowledgeable about health benefits – a ‘*capability*’ component – they would benefit from education at the level of how to integrate a healthier lifestyle in the context of their workplace. By empowering workers and employers, ‘*opportunities*’ emerge in places and situations that may have seemed previously unrealistic. The education processes should also be directed to developing autonomous ‘*motivation*’ towards healthy behaviour [19]. Finally, providing accessible ways to measure the resulting healthy ‘*behaviours*’ is critical in this rationale. For example, in the physical activity context, wearables that track movement can provide a wealth of data for health promotion [20].

## Limitations

Despite limited access to the study population due to security control measures, the sample was sufficient to provide robust formative information for developing workplace health promotion interventions.

## Supplementary Information

**Additional file 1.** Interview Guide.

**Additional file 2.** COREQ (COnsolidated criteria for REporting Qualitative research) Checklist.

## Data Availability

The information collected from the study participants is intimate and at times participants expressed concerns regarding work conditions, indicating the vulnerability to employment. As such, the data collected are not publicly available.

## Abbreviations

LMIC: Low- and middle-income countries
NCD: Non-communicable diseases
