# Supplementary material for: Pharmaceutical workers’ perceptions of physical activity and healthy eating: a qualitative study
Source: BMC Res Notes. 2021 Sep 8;14:350. doi: 10.1186/s13104-021-05765-8 (PMC8425005; doi:10.1186/s13104-021-05765-8)
Supplement: Supplementary file 1 — Additional file 1. Interview Guide. [file 13104_2021_5765_MOESM1_ESM.pdf]

Additional file 1 Interview guide

Pharmaceutical workers' perceptions of physical activity and healthy eating: a qualitative study

Questions concerning physical activity behaviours of the participants

Understanding physical activity in general

- What, in your opinion, is considered as physical activity?
    - What does this mean to you?
- What words would you use to describe the difference in physical activities that adults can do?
- What is the value of physical activity for adults in your own opinion?

Pattern of physical activity during vocation

- Do you think that workers can be physically active at work?
- Do you think that men are more active than women at work?
- What strategies could be used to increase physical activity during work?
- What is your opinion on increasing physical activity during commuting to work?
- What role should management having in increasing physical activity at work?

Understanding sedentary behaviour

- Do you sit while you are at work?
- What is your opinion on breaking up sitting time during work?
- What strategies could be used to reduce or interrupt sitting time at work?
- What strategies could be used to reduce or interrupt sitting time during breaks?
- What strategies could be used to reduce or interrupt sitting time during public transport?

Questions concerning dietary habits of the participants

- What in your opinion, is considered a healthy diet?
    - What in your opinion would be considered an unhealthy diet?
    - What foods and meals could be considered as healthy?
    - What kinds of foods might be considered as unhealthy?
- Do you feel that people are eating a healthy diet during working hours?
    - What do you think?

- Do you think that the company should have a role in promoting healthy eating during working hours?
    - What do you think about this?
- What should management's role be to promote healthy eating at work?

Thank you for participating in the focus group discussion. Your opinion on physical activity behaviours and diet are important.
