# Supplementary material for: Pharmaceutical workers’ perceptions of physical activity and healthy eating: a qualitative study
Source: BMC Res Notes. 2021 Sep 8;14:350. doi: 10.1186/s13104-021-05765-8 (PMC8425005; doi:10.1186/s13104-021-05765-8)
Supplement: Supplementary file 2 — Additional file 2. COREQ (COnsolidated criteria for REporting Qualitative research) Checklist. [file 13104_2021_5765_MOESM2_ESM.pdf]

Additional file 2 COREQ (COnsolidated criteria for REporting Qualitative research) Checklist

Topic	Item number	Guide questions/description	Reported on
**Domain 1: Research team and reflexivity**			
Personal characteristics			
Interviewer/facilitator	1	Which author/s conducted the interview or focus group?	Methods, paragraph describing data collection)
Credentials	2	What were the researcher's credentials?	Methods, paragraph describing data collection
Occupation	3	What was their occupation at the time of the study?	Methods, paragraph describing data collection
Gender	4	Was the researcher male or female?	Methods, paragraph describing data collection
Experience and training	5	What experience or training did the researcher have?	Methods, paragraph describing data collection
Relationship with participants			
Relationship established	6	Was a relationship established prior to study commencement?	Methods, paragraph describing data collection
Participant knowledge of the interviewer	7	What did the participants know about the researcher? e.g. personal goals, reasons for doing the research	Methods, paragraph describing data collection
Interviewer characteristics	8	What characteristics were reported about the inter viewer/facilitator? e.g. Bias, assumptions,	Methods, paragraph describing data collection

		reasons and interests in the research topic	
**Domain 2: Study design**			
Theoretical framework			
Methodological orientation and Theory	9	What methodological orientation was stated to underpin the study? e.g. grounded theory, discourse analysis, ethnography, phenomenology, content analysis	Thematic content analysis (Methods, paragraph describing data analysis)
Participant selection			
Sampling	10	How were participants selected? e.g. purposive, convenience, consecutive, snowball	Participants were recruited purposively (Methods, paragraph describing setting and sample)
Method of approach	11	How were participants approached? e.g. face-to-face, telephone, mail, email	Participants were recruited by telephone
Sample size	12	How many participants were in the study?	28
Non-participation	13	How many people refused to participate or dropped out? Reasons?	No participants dropped out.
Setting			
Setting of data collection	14	Where was the data collected? e.g. home, clinic, workplace	Workplace (Methods, paragraph describing setting)
Presence of non-participants	15	Was anyone else present besides the	No.

		participants and researchers?	
Description of sample	16	What are the important characteristics of the sample? e.g. demographic data, date	Age, gender, job type, years worked at the company (Results section, paragraph 1)
Data collection			
Interview guide	17	Were questions, prompts, guides provided by the authors? Was it pilot tested?	See Additional file 1
Repeat interviews	18	Were repeat inter views carried out? If yes, how many?	N/A
Audio/visual recording	19	Did the research use audio or visual recording to collect the data?	Data were audio recorded using a digital recorder (Methods, data analysis section)
Field notes	20	Were field notes made during and/or after the interview or focus group?	No
Duration	21	What was the duration of the inter views or focus group?	45-60 minutes
Data saturation	22	Was data saturation discussed?	Yes
Transcripts returned	23	Were transcripts returned to participants for comment and/or correction?	No
**Domain 3: analysis and findings**			
Data analysis			
Number of data coders	24	How many data coders coded the data?	3

Description of the coding tree	25	Did authors provide a description of the coding tree?	No, however the coding were informed by the interview guide initially and coding was continuously developed.
Derivation of themes	26	Were themes identified in advance or derived from the data?	Themes were identified from the data.
Software	27	What software, if applicable, was used to manage the data?	Atlas.ti was used to manage the data.
Participant checking	28	Did participants provide feedback on the findings?	No.
Reporting			
Quotations presented	29	Were participant quotations presented to illustrate the themes/findings? Was each quotation identified? e.g. participant number	Yes. (see Table 1 and Table 2)
Data and findings consistent	30	Was there consistency between the data presented and the findings?	Yes (see results section).
Clarity of major themes	31	Were major themes clearly presented in the findings?	Yes (see results section).
Clarity of minor themes	32	Is there a description of diverse cases or discussion of minor themes?	Yes (see discussion section where major and minor themes are interpreted).

Developed from: Tong A, Sainsbury P, Craig J. Consolidated criteria for reporting qualitative research (COREQ): a 32-item checklist for interviews and focus groups. *International Journal for Quality in Health Care*. 2007. Volume 19, Number 6: pp. 349 – 357
